# Opioid poisoning during pregnancy: prevalence, characteristics, and neonatal outcomes

**DOI:** 10.1007/s00737-022-01260-6

**Published:** 2022-08-19

**Authors:** Erin Kelty, Anwyn Pyle, David B. Preen

**Affiliations:** 1 The School of Population and Global Health, the University of Western Australia, 35 Stirling Highway, Crawley, WA Australia; 2 The Department of Health, Government of Western Australia, East Perth, WA Australia

**Keywords:** Maternal health, Opioid overdose, Perinatal outcomes

## Abstract

While it has been postulated that opioid poisoning during pregnancy may cause adverse maternal and neonatal outcomes, the harm associated with opioid poisoning during pregnancy has not been robustly examined. Pregnant women admitted to hospital or presenting to the emergency department (ED) in Western Australia (WA) with a diagnosis of opioid poisoning were identified by linking state midwifery records with hospital and ED administrative data. Maternal and neonatal outcomes were compared with opioid poisoning that occurred in the 12 months prior to conception or the 12 months following birth. Between 2003 and 2018, 57 neonates were born to women who had experienced opioid poisoning during pregnancy (14.1 per 100,000 births) in WA. The incidence of opioid poisoning in the year prior to pregnancy (IRR: 3.04, 95%CI: 2.30, 4.02) and the year following pregnancy (IRR: 1.96, 95%CI: 1.46, 2.64) was significantly higher than during pregnancy. Opioid poisoning during pregnancy was less likely to involve multiple substances and be intentional (rather than accidental). Neonatal conditions associated with in utero hypoxia were significantly less common in neonates born to women who experience opioid poisoning prior to pregnancy compared with during pregnancy (OR: 0.17, 95%CI: 0.04, 0.80). Opioid poisoning in pregnancy was not associated with an increased risk of other serious adverse neonatal outcomes. Opioid poisoning during pregnancy is uncommon and less likely to be intentional and involve multiple substances. Opioid poisoning during pregnancy is likely associated with an increased risk of conditions associated with in utero hypoxia.

## Introduction

While pregnancy is often a substantial motivator to cease opioid use, it is estimated that 0.2 to 0.8% of women will use opioids during pregnancy (Kozhimannil et al. 2017; Whiteman et al. 2014). Opioid use can significantly complicate pregnancy, contributing to poor maternal and neonatal health outcomes, including neonatal opioid withdrawal syndrome (NOWS), low birth weight, pre-term birth, and stillbirth (Kelty and Hulse 2017b; Maeda et al. 2014).

One of the major risks associated with opioid use and misuse is opioid poisoning. Opioid poisoning can adversely affect both maternal and neonatal health, as opioids depress respiratory function, leading to oxygen desaturation and hypoxia, and in extreme cases, cardiopulmonary arrest and death (Kiyatkin 2019). Additionally, maternal oxygen deprivation can also cause fetal hypoxia (Kingdom and Kaufmann 1997). While fatal opioid poisoning during pregnancy is rare, poisoning involving opioids is estimated to account for 11.4% of maternal deaths during pregnancy (Metz et al. 2016; Schiff et al. 2018). Reversal of opioid poisoning using naloxone can cause severe withdrawal symptoms affecting both the mother and neonate (Blandthorn et al. 2018). In extreme circumstances, severe in utero opioid withdrawal may result in fetal seizures and death (Blandthorn et al. 2018).

There is an absence of robust information on the characteristics and outcomes associated with opioid poisoning during pregnancy. At present, much of our understanding of opioid poisoning during pregnancy is largely reliant on case studies, postulated outcomes, and extrapolation from non-pregnancy research. The aims of this study were to (1) estimate the prevalence of opioid poisoning in pregnant women in Western Australia (WA); (2) investigate the characteristics of opioid poisoning during pregnancy, including the involvement of prescription opioids; and (3) examine neonatal harm associated with opioid poisoning during pregnancy.

## Methods

### Study cohort and data sources

The study included a retrospective cohort of live and stillborn neonates born in WA between 1 January 2003 and 31 December 2018 and their mothers, as ascertained from the statutory Midwives Notification System (MNS). The MNS contains all neonates born in WA of at least 20 weeks gestation or at least 400 g birth weight (if gestational age is unknown). In combination with the MNS, administrative hospital and emergency department (ED) records were used to identify women who were admitted to hospital or presented to ED with a diagnosis of opioid poisoning (1) during pregnancy, (2) in the year prior to conception, or (3) the year after birth. Both the hospital and ED data included diagnostic fields coded using the International Statistical Classification of Diseases and Related Health Problems 10th Revision, Australian Modification (ICD-10-AM). For hospital data, this included a primary diagnosis, a co-diagnosis, and up to 20 co-diagnoses fields, while for ED, only a single diagnosis field (recorded as the reason for presentation) was used. Opioid poisoning was identified using ICD-10-AM codes T40.0–T40.4 and T40.6. Fatal opioid poisoning was identified from the WA Death Register. The WA Death Register includes cause of death codes using ICD-10 coding and a flag for women who had been pregnant within 6 weeks of the death. Hospital, ED, and mortality data were available for the period 1 Jan 2002 to 31 December 2019. Data linkage was performed by the WA Data Linking Branch using probabilistic matching of patient names and other identifiers (Holman et al. 1999).

### Prevalence of opioid poisoning in pregnancy

The prevalence of poisoning was calculated for each of the three periods (prior to pregnancy, during pregnancy, and post pregnancy) as a proportion of the total number of neonates born in WA during the study period. For the timing of pregnancy, the estimated date of conception was calculated using the date of birth and the estimated length of gestation (provided within the MNS data).

As estimates of prevalence did not include pregnancies that that did not progress past 20 weeks, pregnancy loss was also examined to help quantify pregnancy loss that may be associated with opioid poisoning. Pregnancies that resulted in abortive outcomes (intentional or unintentional pregnancy loss prior to 20 weeks gestation) were identified from hospital and ED data using ICD-10-AM codes O00-O08. Pregnancies with abortive outcomes were deemed likely associated with opioid poisoning if they occurred within 28 days of an admission to hospital or an ED presentation with an opioid poisoning diagnosis. Pregnancies with abortive outcomes were also examined by type of abortive outcomes; this included ectopic pregnancy (ICD-10: O00), hydatidiform mole (O01), other abnormal products of conception (O02), spontaneous abortion (O03), and medical abortion (O04).

### Opioid poisoning characteristics

Characteristics of opioid poisoning were ascertained from assigned ICD-10-AM codes present in the hospital data. These included the presence of alcohol (ICD-10-AM: T51.0), benzodiazepines (T42.4), stimulants (T40.5, T40.8, T40.9, and T43.6) and cannabis (T40.7), accidental poisoning (X40–X49), intentional poisoning (X60–X69), and poisoning of unknown intent (Y10–Y19). The potential involvement of schedule 8 opioids in the opioid poisoning was ascertained using data from the Monitoring of Drugs of Dependence System. Schedule 8 drugs are a class of drugs with demonstrated efficacy and therapeutic benefit but are associated with an increased risk of misuse, abuse, and dependence. Schedule 8 opioids include prescription opioids for the treatment of opioid use disorders (e.g., methadone and buprenorphine) and for the treatment of pain (e.g., oxycodone, morphine, fentanyl, hydrocodone, and buprenorphine). Opioid poisoning events were considered to have been potentially associated with a schedule 8 opioid, if one has been dispensed in the month prior.

### Neonatal harms

The primary neonatal outcomes of interest were intrauterine hypoxia (ICD-10: P20), hypoxic ischemic encephalopathy (P91.6), and neonatal seizures (P90). They were examined as a combined outcome, as each included diagnosis was uncommon but could be associated with oxygen deprivation that may occur during an opioid poisoning. Identification of the primary outcome was done using data from the Hospital Morbidity Data Collection.

Additional neonatal outcomes examined included birth weight, gestational age at birth, Apgar score, resuscitation required, perinatal mortality, length of neonatal hospital stay and admission to the neonatal special care unit, and diagnosis of fetal distress, neonatal abstinence syndrome (NAS), and infant respiratory distress syndrome (IRDS). Data pertaining to the secondary outcomes were obtained from the Midwives Notification Scheme and the Hospital Morbidity Data Collection.

### Statistical analysis

Descriptive statistics were performed, with numbers and percentages presented for binary and categorical variables and mean, standard deviations, median, and interquartile range presented for continuous variables.

A multilevel mixed-effects generalized linear model, using a Poisson model, was used to compare the prevalence of opioid poisoning during pregnancy to the prevalence in the year before and after pregnancy. Univariable generalized linear models were also used to compare characteristics of the opioid poisonings, including the presence of alcohol and other substances for poisoning, the intent of the poisoning, and the presentation to hospital and/or ED.

In comparing outcomes in neonates born to the three groups, pregnancies resulting in multiple births (i.e., twins and triplets) and subsequent pregnancies were excluded. This was done as multiple births are associated with a higher risk of neonatal complications, and the inclusion of multiple pregnancies per mother may introduce bias. Comparisons between the three groups were performed using univariable generalized linear models.

All analyses were performed using StataMP version 15, with significance set at the *p* < 0.05 level.

### Ethics and consent

The authors assert that all procedures contributing to this work comply with the ethical standards of the relevant national and institutional committees on human experimentation and with the Helsinki Declaration of 1975, as revised in 2008. All procedures involving human subjects/patients were approved by the WA Department of Health Human Research Ethics Committee (RGS0000003029, 10 May 2019) and the University of Western Australia Human Research Ethics Committee (RA/4/20/5530, 18 June 2019). The study protocol was reviewed by the WA Data Linkage Branch and the data custodians. A waiver of consent was approved by both ethics committees, as the study met the criteria set out in the national statement on ethical conduct in human research (2007).

## Results

### Prevalence of opioid poisoning

Between 2003 and 2018, 404,240 neonates were born in Western Australia. Of these, 57 were born to women who attended hospital or ED with a diagnosis of opioid poisoning during pregnancy (14.1 neonates per 100,000 births). Trimester one exposure occurred in 18 neonates (32.7%), trimester two exposure in 15 neonates (27.3%), and trimester three exposure in 23 neonates (41.8%). Additionally, 14 pregnancies with abortive outcomes occurring within 28 days of an opioid poisoning were identified, including 10 medical abortions (71.4%). Fatal opioid poisoning was identified as a cause of death in one pregnant woman (< 20 weeks gestation) and in eight women in the year following pregnancy. The prevalence of opioid poisoning in the years prior to pregnancy (IRR: 3.02, 95%CI: 2.27, 4.02) and the year following pregnancy (IRR: 1.90, 95%CI: 1.40, 2.56) were significantly higher than the prevalence of opioid poisoning during pregnancy (Table 1).

**Table 1:** Number of opioid poisonings resulting in hospital admission or emergency department presentation, occurring prior to, during, or following pregnancy in Western Australian women who gave birth between 2003 and 2018

	Pregnancy^a^	Pre-pregnancy	Post-pregnancy
Number of women	56	231	138
Number of pregnancies^b^	56	236	143
Number of neonates	57	238	146
Number of opioid poisoning events	58	254	159
Opioid poisoning per 100,000 person-years	20.7	63.8	39.9

^a^Not including pregnancies resulting in pregnancy loss prior to 20 weeks of gestation

^b^Women may have given birth multiple times during the study period

### Opioid poisoning characteristics

Opioid poisoning occurring prior to pregnancy was significantly more likely to involve polysubstance use compared with opioid poisoning occurring during pregnancy (Table 2). Although, there was no significant difference in the percentage of opioid poisoning involving polysubstance occurring during pregnancy and those post-pregnancy. The percentage of opioid poisoning in which the women was prescribed an opioid for pain or an opioid for an opioid use disorder in the month prior was also not significantly different for women who experienced opioid poisoning during pregnancy compared with pre- or post-pregnancy. There was no significant difference between pregnancy opioid poisoning and pre- or post-pregnancy opioid poisoning in terms of hospital admissions and/or ED presentations. Opioid poisoning occurring during pregnancy was significantly less likely to be intentional compared with opioid poisoning occurring pre- or post-pregnancy.

**Table 2:** Characteristics of opioid poisonings associated with hospital admission or ED presentation, occurring prior to, during, or following pregnancy in Western Australian women who gave birth between 2003 and 2018

	Pregnancy	Pre-pregnancy		Post-pregnancy	
	*N* (%)	*N* (%)	OR (95%CI)	*N* (%)	OR (95%CI)
Substance involved					
Polysubstance^a^	7 (12.1%)	73 (28.7%)	2.94 (1.27, 6.78)	35 (22.0%)	2.06 (0.87, 4.93)
Alcohol	2 (3.5%)	33 (13.0%)	4.18 (0.97, 17.95)	14 (8.8%)	2.70 (0.60, 12.28)
Benzodiazepines	4 (6.7%)	44 (16.7%)	2.75 (0.95, 8.00)	21 (12.4%)	1.83 (0.60, 5.63)
Stimulants	1 (1.7%)	8 (3.2%)	–^b^	4 (2.5%)	–^b^
Cannabis	1 (1.7%)	1 (0.4%)	–^b^	1 (0.6%)	–^b^
Presentation					
ED only	8 (13.8%)	50 (19.7%)	Reference	43 (27.0%)	Reference
ED and hospital	9 (15.5%)	30 (11.3%)	0.68 (0.30, 1.54)	18 (11.3%)	0.44 (0.19, 1.03)
Hospital only	41 (70.7%)	174 (68.5%)	0.53 (0.19, 1.53)	98 (61.6%)	0.37 (0.12, 1.12)
Intent					
Accidental	21 (36.2%)	37 (14.6%)	Reference	19 (12.0%)	Reference
Intentional	20 (34.5%)	149 (58.7%)	4.23 (2.08, 8.60)	78 (49.1%)	4.31 (1.95, 9.51)
Unknown	17 (29.3%)	68 (26.8%)	2.27 (1.07, 4.83)	62 (39.0%)	4.03 (1.77, 9.16)
Dispensed in the month prior					
Opioids for OUD^c^	7 (12.1%)	15 (5.9%)	0.105	14 (8.8%)	0.473
Opioids for pain^d^	1 (1.7%)	7 (2.8%)	–^b^	3 (1.9%)	–^b^

^a^Only includes alcohol, benzodiazepines, stimulants and cannabis

^b^Number too small for statistical analysis

^c^Methadone, buprenorphine, or buprenorphine and naloxone

^d^Schedule 8 pain medications including morphine, oxycodone, fentanyl, hydromorphone, buprenorphine (for pain)

### Characteristics of mothers

Women who experienced opioid poisoning during pregnancy were not significantly different to those who experience it in the year before or after pregnancy in terms of maternal age, number of previous pregnancies, or smoking status (Table 3). The use of methadone or buprenorphine during pregnancy was more common in women who had experienced opioid poisoning during pregnancy compared with those who experienced opioid poisoning post-pregnancy. Although, there was no difference between pre- and during pregnancy opioid poisonings, in terms of the use of methadone of buprenorphine.

**Table 3:** Characteristics of women who experienced opioid poisoning during pregnancy, in the year prior to pregnancy, or the year following pregnancy in Western Australian women who gave birth between 2003 and 2018

	Pregnancy	Pre-pregnancy	Preg v pre: *p* value	Post-pregnancy	Preg v post: *p* value
Number	55	222	–	104	–
Maternal age, mean ± sd	27.6 ± 6.2	27.8 ± 6.4	0.866	27.5 ± 5.6	0.938
Previous pregnancies, median (IQR)	2 (1, 3)	2 (1, 3)	0.779	2 (1, 4)	0.090
Smoke, *n* (%)	27 (49.1%)	107 (48.2%)	0.973	51 (49.0%)	0.847
Metropolitan residence, *n* (%)	47 (85.5%)	193 (86.9%)	0.772	91 (87.5%)	0.717
OUD agonist during pregnancy^a^, *n* (%)	8 (14.5%)	25 (11.3%)	0.170	8 (8.7%)	**0.013**
Opioids prescribed in pregnancy for pain^b^, *n* (%)	0 (0.0%)	5 (2.3%)	0.502	4 (2.9%)	0.178
Caesarean section, *n* (%)	19 (34.6%)	63 (28.4%)	0.371	33 (31.7%)	0.719

Bold *p* values indicate *p* < 0.05

*IQR* interquartile range, *n* number, *OUD* opioid use disorder, *sd* standard deviation

^a^Methadone and buprenorphine

^b^Prescribed schedule 8 opioids for pain relief during pregnancy, e.g., oxycodone, fentanyl, morphine

### Neonatal outcomes

The primary neonatal outcome, which comprised three conditions associated with in utero oxygen deprivation, was significantly less common in neonates born to women who experience opioid poisoning prior to pregnancy compared with during pregnancy (OR: 0.17, 95%CI: 0.04, 0.80) (Table 4). However, there was no significant difference between neonates born to women who had experienced opioid poisoning post-pregnancy compared with those born to women who experienced opioid poisoning during pregnancy (OR: 0.38, 95%CI: 0.08, 1.76). In all babies born between 2003 and 2018 in WA, 0.9% (*n* = 3472) were diagnosed with the combined outcomes.

**Table 4:** Characteristics and outcomes for neonates born between 2003 and 2018 to Western Australian women who presented to hospital and/or emergency department for opioid poisoning during pregnancy or in the year prior or post pregnancy

	Pregnancy	Pre-pregnancy	Preg v pre: *p* value	Post-pregnancy	Preg v post: *p* value
Number	55	222	–	104	–
Sex (male), *n* (%)	27 (49.1%)	111 (50.0%)	0.904	52 (50.0%)	0.913
Combined outcome^a^	4 (7.3%)	3 (1.4%)	**0.025**	3 (2.9%)	0.215
Birth weight (g), mean ± sd	3,047 ± 582	3,053 ± 748	0.950	3,142 ± 750	0.433
Low birth weight, *n* (%)	8 (14.6%)	40 (18.0%)	0.543	9 (8.7%)	0.258
Gestation (weeks), mean ± sd	37.7 ± 2.3	37.8 ± 3.0	0.742	37.4 ± 3.1	0.587
Pre-term, *n* (%)	9 (16.4%)	31 (14.0%)	0.651	25 (25.0%)	0.264
Fetal distress, *n* (%)	11 (20.0%)	39 (17.6%)	0.675	13 (12.5%)	0.213
Apgar score < 7 at 5 min, *n* (%)	7 (12.7%)	15 (6.8%)	0.149	7 (6.7%)	0.211
Resuscitation required, *n* (%)	22 (40.0%)	86 (38.7%)	0.864	37 (35.65)	0.583
Major resuscitation required^b^, *n* (%)	12 (21.8%)	35 (15.8%)	0.287	14 (13.5%)	0.179
IRDS, *n* (%)	5 (9.1%)	19 (8.6%)	0.900	7 (6.7%)	0.593
NAS, *n* (%)	15 (27.3%)	31 (14.0%)	0.185	17 (16.4%)	**0.030**
Stillborn, *n* (%)	0 (0.0%)	5 (2.3%)	–^c^	2 (1.9%)	–^c^
Length of stay, median (IQR)	4 (3, 7)	4 (2, 5)	0.193	4 (2, 7)	0.915
Special care admission, *n* (%)	16 (29.1%)	57 (26.3%)	0.673	33 (32.4%)	0.674
Threatened abortion, *n* (%)	0 (0.0%)	7 (3.2%)	–^c^	3 (2.9%)	–^c^
Threatened pre-term labor	4 (7.3%)	11 (5.0%)	0.499	6 (5.8%)	0.711

Bold *p* values indicate *p* < 0.05

*IQR* interquartile range, *IRDS* infant respiratory distress syndrome, *NAS* neonatal abstinence syndrome, *n* number

^a^The combined outcome included in utero hypoxia, hypoxic ischemic encephalopathy, and neonatal seizures

^b^Resuscitation involving ventilation and cardiac compressions

^c^Numbers too small for statistical analysis

## Comment

### Principal findings

Opioid poisoning during pregnancy was rare, affecting only 14.1 per 100,000 neonates born in WA between 2003 and 2018 and only a single related death over the study period. Opioid poisoning during pregnancy was less common than opioid poisoning in the year prior to conception and the year following birth, with more than twice as many women experienced opioid poisoning in the year prior to pregnancy compared with the year following pregnancy. Additionally, opioid poisoning during pregnancy was less likely to involve polysubstance use and was more likely to be deemed accidental.

Opioid poisoning during pregnancy was not associated with pregnancy loss after 20 weeks of gestation, with only a single perinatal death occurring in women who had been poisoned during pregnancy. While there were a number of pregnancy losses before 20 weeks, occurring within 28 days of opioid poisoning, the majority of these were medical terminations rather than spontaneous abortion. There was initial evidence to suggest opioid poisoning during pregnancy is associated with an increased risk of neonatal outcomes associated with in utero oxygen deprivation (intrauterine hypoxia, hypoxic ischemic encephalopathy, and neonatal seizures). However, the outcome was only identified in four neonates, making comparisons between groups difficult.

### Results in the context of what is known

Compared with non-pregnant patients, the ratio of non-fatal to fatal opioid poisoning in pregnant women was much higher than has been previously observed (58 non-fatal compared with one fatal). For example, in a study of patients with an opioid use disorder, 5.70 non-fatal opioid poisonings were observed for every fatal opioid poisoning (Kelty and Hulse 2017a). This in combination with the lower percentage of intentional and polysubstance opioid poisoning may reflect pregnancy being a substantial motivator for reducing opioid and other substance use in women. In other studies, pregnancy has also been shown to be associated with a reduced risk of suicide (Marzuk et al. 1997; Appleby 1991). This may reflect increased optimism and purpose observed during pregnancy, as well as increases support and services provision.

Despite the small sample size, there was no evidence to suggest opioid poisoning in pregnancy is associated with high rates of stillbirth, which is a common concern. Investigation of pregnancy loss (< 20 weeks) was substantially more difficult, as pregnancy loss may take place before a patient is aware they are pregnant, and that pregnancy terminations and/or care can occur outside of the hospital setting. In this study, in pregnant women who had experienced opioid poisoning, pregnancy loss was most frequently a termination, rather than a spontaneous abortion. It is likely that the use of opioids and other substances factored into the decision to terminate the pregnancy. This is consistent with the higher proportion of medical abortions in women who use illicit opioids compared with non-opioid using women (Cornford et al. 2015).

While there was evidence to suggest the opioid poisoning may cause neonatal hypoxia in some cases, opioid poisoning during pregnancy was not a substantial contributor to intrauterine hypoxia, hypoxic ischemic encephalopathy, or neonatal seizures, with approximately 0.1% of all diagnoses occurring in neonates exposed to opioid poisoning in utero. Otherwise, opioid poisoning did not appear to contribute to the poor outcomes associated with opioid use during pregnancy, with neonatal outcomes typically not differing significantly in neonates born to women who experienced opioid poisoning in the year prior to conception or the year following birth. However, it is worth noting that their outcome is likely to be inferior compared with the neonates born to women with no history of opioid use.

Other neonatal outcomes did not differ between the three groups, with the exception of NAS. The proportion of neonates diagnosed with NAS was significantly higher in neonates born to mothers who experienced opioid poisoning during pregnancy compared to those born to women who had experienced opioid poisoning in the year following birth. This may reflect the higher percentage of neonates exposed to either methadone or buprenorphine in utero, with women likely supported onto an opioid pharmacotherapy following opioid poisoning.

### Limitations

There were a number of limitations in this study, primarily associated with the use of linked administrative data and the available sample size. The study was only able to capture opioid poisoning that was admitted to hospital or presented to ED and pregnancy loss that occurred in the same setting. The inability to identify events that occurred outside of hospital means that prevalence is underestimated.

While the study included a whole-population cohort of all births in WA over a 16-year period, a low occurrence of opioid poisoning during pregnancy resulted in a small sample size in that group. This affected the examination of neonatal outcomes, particularly less common outcomes such as perinatal mortality. Similarly, only a single case of fatal opioid poisoning was identified. Thus, it was not possible to examine the characteristics of fatal opioid poisoning during pregnancy. As such, further work on a larger cohort is warranted. Despite these limitations, this study focuses on an important avenue for future research and begins to bridge a substantial void in the current literature on opioid poisoning during pregnancy.

## Conclusion

Opioid poisoning during pregnancy is not common, compared with opioid use prior to or after pregnancy, with pregnancy likely a substantial motivator for opioid discontinuation. Characteristics of opioid poisoning in pregnancy were also different in terms of the lack of involvement of multiple substances and intent. Preliminary evidence suggested an increased risk of neonatal conditions associated with oxygen deprivation in utero. However, further research on a much larger population is required.

## Data Availability

Data utilized in this study is not publically available due to data custodian concerns surrounding participant confidentiality.

## Code availability

Code is available upon request.
